# The Relationship Between DNA Mismatch Repair Status and Clinicopathologic Characteristics in Colon Cancer

**DOI:** 10.5152/tjg.2024.23366

**Published:** 2024-09-01

**Authors:** Mehmet Doğan, Mehmet Kılıç, Hayriye Tatlı Doğan

**Affiliations:** 1 Department of Pathology, SBÜ Dr. Abdurrahman Yurtaslan Ankara Education and Research Hospital, Ankara, Türkiye; 2 Department of Surgery, Eskişehir Osmangazi University Faculty of Medicine, Eskişehir, Türkiye; 3 Department of Pathology, Ankara Yıldırım Beyazıt University Faculty of Medicine, Ankara Bilkent City Hospital, Ankara, Türkiye

**Keywords:** Mismatch repair, Lynch, colorectal cancer, BRAF

## Abstract

**Background/Aims:**

DNA mismatch repair (MMR) proteins are essential for repairing genetic mutations that occur during DNA replication. Deficiency of MMR proteins results in a phenotype called microsatellite instability (MSI), which occurs in Lynch syndrome as well as sporadic colorectal cancers (CRC), and it is associated with several clinicopathological features. We aimed to investigate the association of the loss of MMR proteins with clinicopathologic considerations in our CRC series.

**Materials and Methods:**

In this retrospective study, DNA MMR protein status in CRC is evaluated in a total of 200 colorectal resection specimens by immunohistochemistry (IHC) for MLH1, MSH2, MSH6 and PMS2 protein expression. The BRAF mutation was investigated by the real-time PCR in cases with loss of MLH1 protein expression. The relationship between MMR status and clinicopathological parameters was investigated statistically.

**Results:**

Loss of MMR protein expression was detected in 26 of 200 CRC cases. The BRAFV600E mutation was detected in 2 of the cases with MLH1 loss and accepted as sporadic. The remaining 24 cases (12%) were identified as Lynch syndrome candidates. There were statistical differences observed regarding the presence of tumor-infiltrating lymphocytes (*P* < .001), Crohn’s-like reaction (*P* = .001), expansile growth (*P* < .001), tumor heterogeneity (*P* < .001), mucinous differentiation (*P* < .001), and presence of metastatic lymph nodes (*P* = .045) between sporadic cases with preserved MMR and Lynch candidates. However, difference in the survival rates between sporadic cases and Lynch candidates was not significant.

**Conclusion:**

Immunohistochemical staining for MMR is a practical method for predicting MSI phenotype as well as Lynch candidates. MMR expression status was found to be associated with certain clinicopathological features some of which also have prognostic significance.

**Main Points**

- DNA mismatch repair (MMR) protein expression and detection of Lynch candidates according to MMR deficiency were evaluated by immunohistochemical staining in colorectal cancers (CRCs).
- MMR protein status in CRC was found to be associated with tumor location and certain histopathological features. There was a significant difference between sporadic cases with preserved MMR and Lynch candidates in terms of proximal colon localization, expansile growth, mucinous differentiation, tumor-infiltrating lymphocytes, Crohn’s-like reaction, tumor heterogeneity, and the presence of metastatic lymph nodes.
- In addition to its relationship with the mentioned histopathological features, MMR protein status is also important to better understand the biological features of the tumor and to predict Lynch candidates.

## Introduction

Colorectal cancer (CRC) is quite common, ranking second in mortality and third in incidence when it comes to types of cancer.^1^ The incidence has been increasing in Latin America, Asia, and Eastern Europe.^2^ The microsatellite instability (MSI) mutational pathway is crucial for carcinogenesis in a somewhat lessened, but essential, proportion of CRCs. Defects in the deoxyribonucleic acid (DNA) mismatch repair (MMR) proteins result in a phenotype called MSI, while the system of MMR is in charge of monitoring and correcting errors presented through the microsatellite regions in DNA.^3^ The core elements of this system are MLH1, MSH2, MSH3, MSH6, and PMS2 proteins, and they function as heterodimers.

About 15% of CRCs show MSI resulting from epigenetic silencing of MLH1 or a germline mutation in one of the MMR genes including MLH1, MSH2, MSH6, or PMS2. Germline mutation in MMR genes is linked to hereditary non-polyposis colorectal cancer (HNPCC) or Lynch syndrome. HNPCC makes up 2%-4% of all CRCs,^4,5^ and can be classified as the most frequently appearing hereditary cancer predisposition syndrome. The BRAF p. (V600E) mutation is detected in as many as 70% of tumors with loss of expression of PMS2 and MLH1 or show signs of MLH1 promoter methylation.^6^ The BRAF p. (V600E) mutation is rarely observed in tumors associated with Lynch syndrome.^7^ This being the case, evidence of the BRAF mutation can certainly show that the dMMR tumor has a sporadic origin.^8^ Both Polymerase Chain Reaction (PCR) based MSI detection and immunohistochemical (IHC) testing are methods of high sensitivity that are utilized in identifying a potentially defective MMR system. They can also help direct clinicians toward genetic testing that is both cost-effective and informative.^9,10^

There are clinical and pathological predictors which reflect MSI phenotype. Proximal colon localization, medullary, signet ring cell or mucinous morphology are found to be related to MSI deficiency. Besides, it can be observed in the literature that there was a significant correlation between tumor-infiltrating lymphocytes and Crohn’s-like reaction.^11,12^

The present study intended to identify the relationship between the MMR deficiency, which is critical in predicting defective MMR and determining Lynch candidates, and histopathological and clinical prognostic parameters in our CRC series.

## Materials and Methods

Two hundred patients who had undergone resection for a colorectal adenocarcinoma were included in this retrospective study. Before the start of the study, ethical approval for the study was received from the Ethics Committee of Ankara Yıldırım Beyazıt University (approval number: 26379996/163, date: December 18, 2013). Patient assent was not required.

The clinical characteristics of the patients were obtained from the hospital database. Data on the patients obtained from medical records included age, sex, localization, tumor–node–metastasis (TNM) stage, distant metastasis, date of diagnosis, and date of death.

To pinpoint the location of the tumor, the colon was divided into 8 regions and these were collapsed into 2 categories: proximal colon (i.e., cecum, ascending colon, hepatic flexure, and transverse colon), and distal colon (i.e., splenic flexure, descending colon, sigmoid, and rectum). The hematoxylin and eosin (H&E)-stained sections of all cases were reviewed by 2 pathologists and the following histological criteria were used to evaluate the tumors.^11^

### Histopathological Features

#### Tumor Grade and Tumor–Node–Metastasis Cancer Stage

For the definition and classification of study variables that included TNM cancer stage and histological grade, the 2019 World Health Organization (WHO) Classification of Tumors of the Digestive System was consulted.

#### Mucinous Differentiation

A mucinous carcinoma was defined by ≥50% mucin. Tumors containing less than 50% mucinous area were classified as having focal mucinous differentiation.

#### Histological Heterogeneity

Tumors were noted to have histological heterogeneity if they demonstrated at least 2 growth patterns distinct from one another.^11^ These did not include mucinous and non-mucinous areas, unless other pattern differences were evident, such as architecture or tumor grade.

#### Tumor Necrosis

All tumors were evaluated to determine whether or not “dirty necrosis” was present, a feature typical of CRC.

#### Growth Pattern of Tumor at Advancing Edge

The advancing edge of the tumor was analyzed to determine the means by which the tumor grew. It could appear as an expansile or pushing pattern, or an infiltrative pattern.

#### Crohn’s-Like Host Response

The advancing edge of the tumor was evaluated for the presence of a Crohn’s-like inflammatory response. A prominent response was defined as a minimum of 3 lymphoid aggregates per section.^11^

#### Tumor-Infiltrating Lymphocytes

Tumor-infiltrating lymphocytes (TILs) were recognized on H&E-stained sections as small mononuclear cells with something reminiscent of a halo. Also counted were the lymphocytes infiltrating between tumor cells. The tumor was scanned at low power to determine which area had the highest number of TILs. After this assessment was made, 5 consecutive 40x fields of a Nikon Eclipse 80i microscope with a Plan objective were obtained. The mean TIL/high-power field for each tumor was subsequently established by dividing the total number of TIL by 5. The cut-off value of >2 TIL/HPF was accepted as TIL positive.^11^

#### Lymph Node Assessment

For the evaluation of the role of dMMR on both adequate and total lymph node count, data of 200 CRC patients were taken into account. A total of ≥12 lymph nodes was used as the cut-off value for the distinction between cancers with adequate and inadequate lymph node retrieval.

### Immunohistochemistry and BRAF Mutation Analysis

One of the tumor blocks indicating the tumor properties best and including tumor area adjacent to normal mucosa or lymphocytic infiltration was selected from the pathology archives retrospectively. Four-micrometer sections were obtained from formalin-fixed, paraffin-embedded tumors. Immunohistochemical studies were carried out manually by the streptavidin biotin method using commercially available kits for MLH-1 (1/100, Cell Marque, Clone G168-728), MSH-2 (1/200, Cell Marque, clone G219-1129), PMS-2 (1/200, Cell Marque, clone EPR3947), and MSH-6 (1/200 ThermoFisher, clone 44). The positive control was nuclear staining in normal mucosa and/or lymphocytic infiltration. An absence of staining within the tumor, with normal expression in the internal control cells, indicated a loss of expression for the protein.

The BRAF mutation was investigated by real-time polymerase chain reaction (PCR) using the Entrogen BRAF V600E mutation kit in cases with loss of MLH1 protein expression was shown by IHC.

### Statistical Analysis

Statistical analysis was performed using the Statistical Package for the Social Sciences version 22.0 software (IBM Corp., Armonk, NY, USA). Patient demographic data and histopathological findings were recorded in spreadsheet software. The necessary error checks and corrections were performed.

The differences between the continuous variables according to the MMR status were investigated using the Mann–Whitney *U*-test. Diagonal tables were used for the differences between categorical variables such as gender, tumor localization, differentiation, presence of lymphocytes, according to the MMR status, and the chi-square (*χ* ^2^) values were calculated. The Kaplan–Meier survival analysis was employed to investigate the impact that MMR status had on the median survival time. The survival time between the groups was investigated using the log-rank (Mantel–Cox) test. A *P* value of <.05 was considered statistically significant.

## Results

A total of 200 patients, 139 (69.5%) were males and 61 (30.5%) were females. The mean age was 65.6 ± 11.66 ± 11.6, with a range of 35 to 93 years. The mean age for women was 66.2 ± 11.0 years, and it was 65.3 ± 11.9 years for men. The location was proximal colon in 67 of the patients and distal colon in 119 of the patients. The location was not specified in 14 patients.

### Relationship Between Mismatch Repair Protein Status and Clinical Features

**Figure 1.**
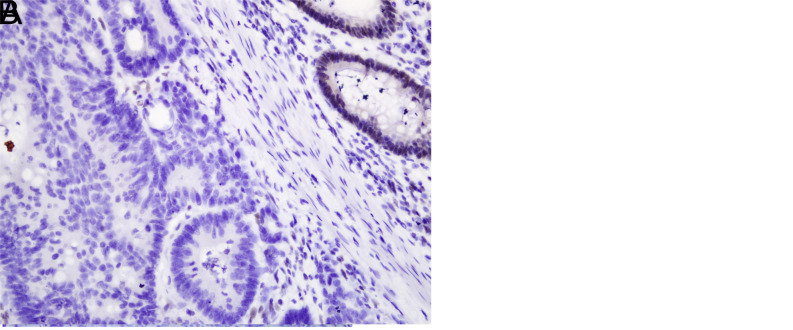
(A) Loss of MLH1 staining in adenocarcinoma compared to adjacent normal colonic epithelium (×400). (B) Loss of PMS2 in colonic adenocarcinoma (×400).

**Figure 2.**
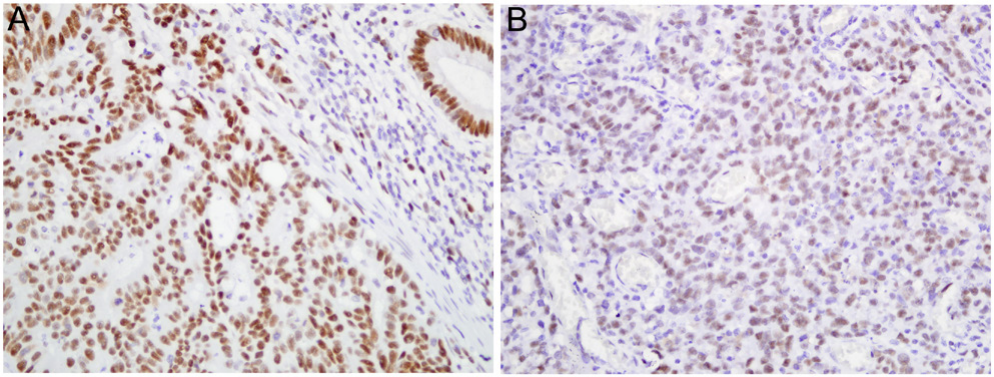
(A) Extensive nuclear staining with MSH6 in colonic adenocarcinoma (×400). (B) Tumor cells showing positive nuclear staining with MSH 2 (×400).

Loss of MMR protein was detected in 13% of patients (n = 26n = 26) by IHC. Loss of MLH1&PMS2, MSH2&MSH6, and isolated MSH6 were observed in 16, 9, and 1 patient, respectively (Figure 1A and 1B). The rest of the cases showed diffuse or at least focal nuclear staining (Figure 2A and 2B). BRAF mutation was detected in 2 of 16 patients with loss of MLH1 and accepted as sporadic CRC. There was no significant difference in the frequency of loss of MMR between males and females. However, loss of MMR was higher in the proximal colon (63.6%) than in the distal colon (36.4%) (*P* = .004). Tumor diameter tended to be higher in patients with MMR protein loss (*P* < .001). The difference between deficient MMR (dMMR) and preserved MMR (pMMR) cases for the presence of lymph node metastasis was considered significant (*P* = .045). The presence of metastatic lymph nodes was found to be more common in MMR-preserved sporadic patients than MMR-deficient ones. The comparison of clinicopathological parameters according to the MMR status is shown in Table 1.

**Figure 3.**
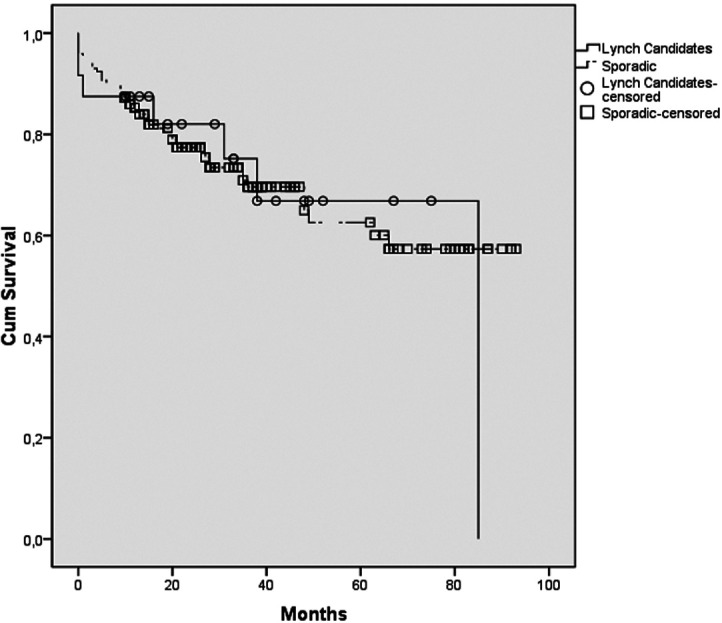
Kaplan–Meier survival curve of the sporadic and Lynch candidates.

There was no relationship between the loss of MMR and distant metastasis, lymphovascular invasion, presence of perineural invasion, and T stage. The median survival in sporadic cases with pMMR and cases with dMMR was 64.6 months and 63 months, respectively. Sporadic cases had an approximately 1.6-month-longer median survival time than dMMR cases, although it did not reach statistical significance (*P* = .978) (Figure 3).

### Relationship Between Mismatch Repair Protein Status and Histopathological Findings

Mucinous differentiation was identified in 62.5% of the patients with loss of MMR and in 19.3% of the patients with preserved MMR. Crohn’s-like reaction, TILs, expansile growth pattern, tumor heterogeneity, and the presence of mucinous differentiation were found to be higher in the patients with dMMR compared to those with pMMR (*P *< .005) (Table 2).

In dMMR cases, the rate of poor differentiation of the tumor was about 3 times higher than that of sporadic cases, indicating a statistically significant difference (*P* < .001). However, there was no significant difference in the rate of dirty necrosis.

The median number of metastatic lymph nodes was similar between pMMR cases and Lynch candidates (*P* = .340). The median number of reactive lymph nodes was higher in the dMMR group than in the sporadic cases (*P* = .013). Similarly, the median number of total lymph nodes was higher in dMMR cases than in the sporadic ones (*P* = .017) (Table 3). However, the difference in the retrieval of ≥12 lymph nodes between the dMMR and sporadic cases was not found to be significant (*P *= .09). In addition, there was no significant difference regarding adequate lymph node retrieval between deficient MMR and sporadic cases in proximal localized tumors (*P* = .227).

## Discussion

This research evaluated MMR protein expression in CRC patients. The incidence of dMMR CRCs in our study was 12%, which was more than that in Eastern countries^13^ but was slightly less than in Western countries.^14^ MMR deficiency predicts Lynch candidates, but germline mutation tests are required for precise diagnosis of Lynch.

Similar to previous studies,^11,15,16^ MMR protein-negative tumors in the Turkish population were proximally located and demonstrated poor histological differentiation. Consistent with the Jenkins et al’s study,^17^ we also found that the presence of mucinous histology, Crohn’s-like inflammatory reaction, and TIL cells were strong predictors of MSI.

There is little reporting in the Turkish population with CRC about the MMR protein expression.

Erdamar et al^18^ reported loss of either MLH1 or MSH2 expression in 32 (43.2%) of 74 patients. They saw no correlation of any significance between MLH1/MSH2 expression and tumor type and size, though a noteworthy relationship was detected between tumor invasion and MSH2 expression.

Tunca et al^19^ selected patients out of families that fell in line with the Amsterdam criteria and were suspected of having HNPCC. They performed PCR-based MSI analysis and IHC analysis of MLH1, MSH2 proteins, and MLH1, MSH2 mutation detection. They found that 10 (35.7%) of 28 patients were MSI-positive. Analysis of MSI-positive tumors also revealed that there was no MMR protein expression showing 100% sensitivity.

Similar to our results, Karahan et al^16^ found a positive correlation between the loss of MLH-1&PMS-2, MSH-2&MSH-6 expressions and the right-colon location, poor and mucinous differentiation of a total of 186 resection materials with CRC.

Several studies showed that dMMR cases tend to have prognoses superior to MMR proficient (pMMR)^20-22^ ones. Sari et al found dMMR 21 (25.3%) out of 83 stage II CRC patients, and TIL-high/dMMR tumors had a better prognosis than TIL-low/proficient MMR tumors.^23^ We noted that the difference in overall survival between sporadic and dMMR cases was not significant.

The number of lymph nodes involved is a crucial prognostic indicator.^24^ Several studies have shown that more lymph nodes are retrieved from right-sided tumors than from left-sided.^25,26^ Structural differences of the blood and lymphatic vessel anatomy are supposedly responsible for this discrepancy.^27^ Various studies have made a connection between the status of MSI and lymph node retrieval.^28-32^ Consistent with prior studies, the data we gathered seem to show a statistically significant difference in the median number of reactive and total lymph nodes retrieved in CRC with Lynch candidates compared to MMR-preserved sporadic tumors.

There can be substantial therapeutic implications in the evaluation of colon cancers for MMR defects. CRC patients, those with MMR-deficient tumors, seem not to benefit from 5-FU-based chemotherapy.^33-35^ In addition, MSI tumors may be more responsive to irinotecan than microsatellite stable ones.^36^ A number of studies have shown that MSI tumors have a more favorable prognosis and are less prone to lymph node and systemic metastasis.^3,15,37^ In the 2010s, MSI-high colorectal tumors were classified as high-grade tumors whereas they are now regarded as relatively low-grade tumors with differing chemosensitivity and response to PD-L1 blockade therapy.^38^

Nowadays, MMR deficiency has become a predictive biomarker for immunotherapy efficiency especially in advanced stage patients, as it reflects the MSI status. It is essential not only for the identification of inherited predisposition to cancer but also for guiding oncologists with treatment choices associated with both chemotherapy and immunotheraphy.^39,40^ According to the National Comprehensive Cancer Network (NCCN) guidelines version 2.2017, Lynch syndrome tumors screening (i.e., IHC for MMR or PCR for MSI) should be conducted for all patients with CRC diagnosed at age ≤70 years, as well as those >70 years that fall in line with the Bethesda guidelines.^41^ Universal MMR or MSI testing is advised for all patients with a personal history of colon cancer. MSI’s function as a genetic indicator of Lynch syndrome has been well documented. Both IHC and PCR-based assays for MSI detection are considered methods of high sensitivity, quite useful in the identification of a defective MMR system, and they can lead clinicians toward genetic testing that is both cost-effective and informative.

The findings such as the age of the patient, the localization of the tumor, and aside from these; the morphological features that indicate MSI phenotype which include mucinous, medullary differentiation, tumor infiltrating lymphocytes, Crohn’s like reaction require the assessment of a pathologist, as well as the direction of patients to the MSI test in medical facilities where IHC or PCR tests cannot be performed because of the cost limitations.^12,42^

The study did have some limitations worthy of note. First, it was retrospective and we were not able to evaluate family history of cancer. Second, we only evaluated MLH1, PMS2, MSH2, and MSH6 protein expression. Additionally, none of the patients were evaluated with PCR-based MSI testing to confirm the findings of IHC. We did not perform MMR gene germline analysis and therefore the exact proportion of hereditary and sporadic cases was unknown. Our investigation on MMR status in CRCs is one of the broadest studies in Türkiye evaluating in detail its relationship with histopathological and clinical findings.

In summary, the rate of MMR deficiency was 12% in this colon cancer series of 200 patients. Proximal colon localization, mucinous morphology, Crohn’s-like reaction, TILs, expansile growth pattern, tumor heterogeneity, and were found to be higher in the patients with dMMR compared to those with pMMR. The presence of lymph node metastasis was higher in patients with pMMR than in those with MMR loss. IHC testing for MMR proteins should be performed to improve the efficiency of the prognostic evaluation of CRC and predicting for Lynch.

Apart from Lynch syndrome, studies detecting relationships between colorectal cancer and genomic polymorphisms in the Turkish population,^43,44^ are also becoming increasingly important.

CRC is a heterogeneous disease and MMR status is a well-known prognostic biomarker. In addition, recent studies that take into account the morphological and prognostic features of the tumor, as well as biological and molecular features such as immune cell infiltration^45^ and post-translational modification,^46^ will allow the discovery of new biomarkers.

## Figures and Tables

**Table 1.** Comparison of the Clinicopathologic Characteristics According to MMR Status

Variable	Unit	Preserved MMR (Sporadic)	Deficient MMR (Lynch Candidate)	Total	*P*
n	n (%)	176 (88%)	24 (12%)	200 (100%)	
Age	Median (IQR)	66.0 (15.0)	64.0 (23.0)	66.0 (16.0)	**.039**
Sex	Female (n, %)	54 (30.7)	7 (29.2)	61 (30.5)	.880
	Male (n, %)	122 (69.3)	17 (70.8)	139 (69.5)	
Tumor size (cm)	Median (IQR)	4.5 (2.5)	7.0 (3.5)	5.0 (2.5)	**<.001**
Tumor stage	T1 (n, %)	3 (1.7)	1 (4.2)	4 (2.0)	.127
	T2 (n, %)	18 (10.2)	0 (0.0)	18 (9.0)	
	T3 (n, %)	118 (67.0)	16 (66.7)	134 (67.0)	
	T4 (n, %)	37 (21.0)	7 (29.2)	44 (22.0)	
Lymph node metastases	No (n, %)	79 (44.9)	16 (66.7)	95 (47.5)	**.045**
	Yes (n, %)	97 (55.1)	8 (33.3)	105 (52.5)	
Pre-op distant metastases	No (n, %)	158 (91.9)	22 (91.7)	180 (91.8)	.974
	Yes (n, %)	14 (8.1)	2 (8.3)	16 (8.2)	
Follow up distant metastases	No (n, %)	92 (52.3)	12 (50.0)	104 (52.0)	.489
	Yes (n, %)	43 (23.5)	4 (16.7)	47 (23.5)	
Lymphovascular invasion	No (n, %)	71 (51.8)	11 (52.4)	82 (51.9)	.962
	Yes (n, %)	66 (48.2)	10 (47.6)	76 (48.1)	
Perineural invasion	No (n, %)	93 (71.5)	18 (90.0)	111 (74.0)	.080
	Yes (n, %)	37 (28.5)	2 (10.0)	39 (26.0)	
Survival	Ex (n, %)	51 (29.1)	7 (29.2)	58 (29.1)	.998
	Alive (n, %)	124 (70.9)	17 (70.8)	141 (70.9)	
Localization*	Distal colon	111 (67.7)	8 (36.4)	119 (64.0)	**.004**
	Proximal colon	53 (32.3)	14 (63.6)	67 (36.0)	
Differentiation	well	55 (31.3)	2 (8.3)	57 (28.5)	**<.001**
	moderate	97 (55.1)	9 (37.5)	106 (53.0)	
	poor	24 (13.6)	13 (54.2)	37 (18.5)	

Values in bold indicate statistical significance.

*Fourteen cases with unknown localization were excluded.

**Table 2.** Histopathological Features According to the Mismatch Repair Status

Variable	Unit	Preserved MMR (Sporadic)	Deficient MMR, BRAF Wild (Candidate for Lynch)	Total	*P*
TILs	No (n, %)	135 (76.7)	8 (33.3)	143 (71.5)	**<.001**
	Yes (n, %)	41 (23.3)	16 (66.7)	57 (28.5)	
Crohn’s-like reaction	No (n, %)	150 (82.5)	14 (58.3)	164 (82.0)	**.001**
	Yes (n, %)	26 (14.8)	10 (41.7)	36 (18.0)	
Dirty necrosis	No (n, %)	96 (54.5)	18 (75.0)	114 (57.0)	.058
	Yes (n, %)	80 (45.5)	6 (25.0)	86 (43.0)	
Growth pattern	Infiltrative (n, %)	159 (90.3)	15 (62.5)	174 (87.0)	**<.001**
	Expansile (n, %)	17 (9.7)	9 (37.5)	26 (9.7)	
Heterogeneity	No (n, %)	175 (99.4)	17 (70.8)	192 (96.0)	**<.001**
	Yes (n, %)	1 (0.6)	7 (29.2)	8 (4.0)	
Mucinous differentiation	Yes (n, %)	34 (19.3)	15 (62.5)	49(24.5)	**<.001**
	No (n, %)	142 (80.7)	9 (37.5)	151 (75.5)	

Values in bold indicate statistical significance. MMR, mismatch repair: TILs, tumor-infiltrating lymphocytes.

**Table 3.** The Median Number of Lymph Nodes According to the Mismatch Repair Status

Variable	Preserved MMR (Sporadic)	Deficient MMR, BRAF Wild (Candidate for Lynch)	*P*
Metastatic lymph node	1.00 (3.00)	0.00 (4.75)	.340
Reactive lymph node	12.00 (12.00)	19.00 (15.25)	**.013**
Total lymph node	14.00 (13.00)	24.50 (17.25)	**.017**

Values in bold indicate statistical significance. MMR, mismatch repair.
